# IgG4-related disease with subcutaneous involvement and the associated diagnostic challenges with MRI

**DOI:** 10.1007/s00256-024-04768-3

**Published:** 2024-07-31

**Authors:** Tomonori Kawasaki, Jiro Ichikawa, Kojiro Onohara, Satoshi Kanno, Masanori Wako, Naofumi Taniguchi, Satoshi Ochiai, Tomoaki Torigoe, Yasuo Yazawa

**Affiliations:** 1 Department of Pathology, Saitama Medical University International Medical Centre, Saitama, Japan; 2 Department of Orthopaedic Surgery, Interdisciplinary Graduate School of Medicine, University of Yamanashi, Yamanashi, Japan; 3 Interdisciplinary Graduate School of Medicine, University of Yamanashi, Yamanashi, Japan; 4 Department of Orthopaedic Surgery, National Hospital Organization (NHO) Kofu National Hospital, Kofu, Yamanashi Japan; 5 Orthopaedic Oncology & Surgery, Saitama Medical University International Medical Centre, Saitama, Japan

**Keywords:** IgG4-related disease, Subcutaneous, MRI, Differential diagnosis, Biopsy, Histopathology

## Abstract

IgG4-related disease is a rare fibroinflammatory disorder characterized by the infiltration of IgG4-rich plasma cells. Herein, we report a case of IgG4-related disease of the subcutaneous tissue with atypical MRI findings and difficulties in the histopathological examination using needle biopsy. Based on the clinical presentation and MRI findings, the patient was diagnosed with a benign myxoid or cystic tumor. Additionally, histopathological findings from a needle biopsy suggested a myxoma. Therefore, the correct diagnosis of IgG4-related disease was not made preoperatively. The resected specimens confirmed IgG4-related disease with an IgG4/IgG ratio > 80%. Previous reports have shown that the MRI findings of IgG4-related disease mimic both malignancy and inflammation; surprisingly, the features of subcutaneous IgG-related disease, including tail sign, unclear border, and heterogeneous enhancement, were similar to those found in sarcoma. Therefore, histopathological findings are needed for a correct diagnosis. Furthermore, careful examination is essential because the neoplasm and inflammation may overlap with IgG4-related disease, and needle biopsy is not fully reflective of the tumor. As is highlighted in the present case, IgG4-related disease is often misdiagnosed; therefore, clinicians should adequately recognize that even if the histopathological findings in biopsy were consistent with those observed in the MRI, misdiagnosis may occur.

## Introduction

IgG4 related disease (IgG4-RD) is a rare systemic fibroinflammatory condition characterized by chronic inflammation and fibrosis [1]. The proposed diagnostic criteria for IgG4-RD are as follows: 1) organ enlargement, masses or nodular lesions, or organ dysfunction; 2) an increase in serum IgG4 levels; and 3) histopathological findings, including lymphoplasmacytic infiltration, storiform fibrosis, obliterative phlebitis, and infiltration of IgG4 cells [1]. Various organs may be involved, and most cases occur in the pancreas; however, it is rarely observed in the subcutaneous tissue [2, 3]. During treatment, careful follow-up is performed in asymptomatic patients; however, in symptomatic patients with organ disorders, steroids are the first choice of treatment. Furthermore, disease-modifying antirheumatic drugs are often added in cases of failure or relapse [4]. Therefore, the diagnosis and treatment of IgG4-RD are rational; however, there have been no suggestions regarding the diagnostic features of imaging findings that distinguish between malignancy, infection, and inflammation, resulting in the diagnosis frequently being difficult [5]. Interestingly, in subcutaneous cases, which is an extremely rare site, the MRI findings were reported to be similar to those of sarcomas, suggesting that biopsy followed by histopathological examination is crucial for the diagnostic process [3, 6]. In addition, even when the diagnosis is performed by histopathological examination, differentiating between malignancy and inflammation can sometimes be difficult, and a small sample size makes the diagnosis even more difficult [1, 7]. In this report, we describe a case of IgG4-RD with rare involvement of the subcutaneous tissue and difficulties associated with the imaging and pathological findings.

## Case report

One year prior to his first visit to our hospital, a 58-year-old man noticed a mass in his lower abdomen. The patient was referred to our hospital for further diagnosis and treatment. The mass was located at the midline of the lower abdomen; it felt hard with no tenderness, swelling, or Tinel’s sign, and had good mobility. The superficial lymph nodes were not palpable, and his body temperature was within the normal range. Blood test results showed a white blood cell count of 9650/uL (3300–8600) and a C-reactive protein level of 0.08 mg/dL (0–0.25).

**Fig. 1:**
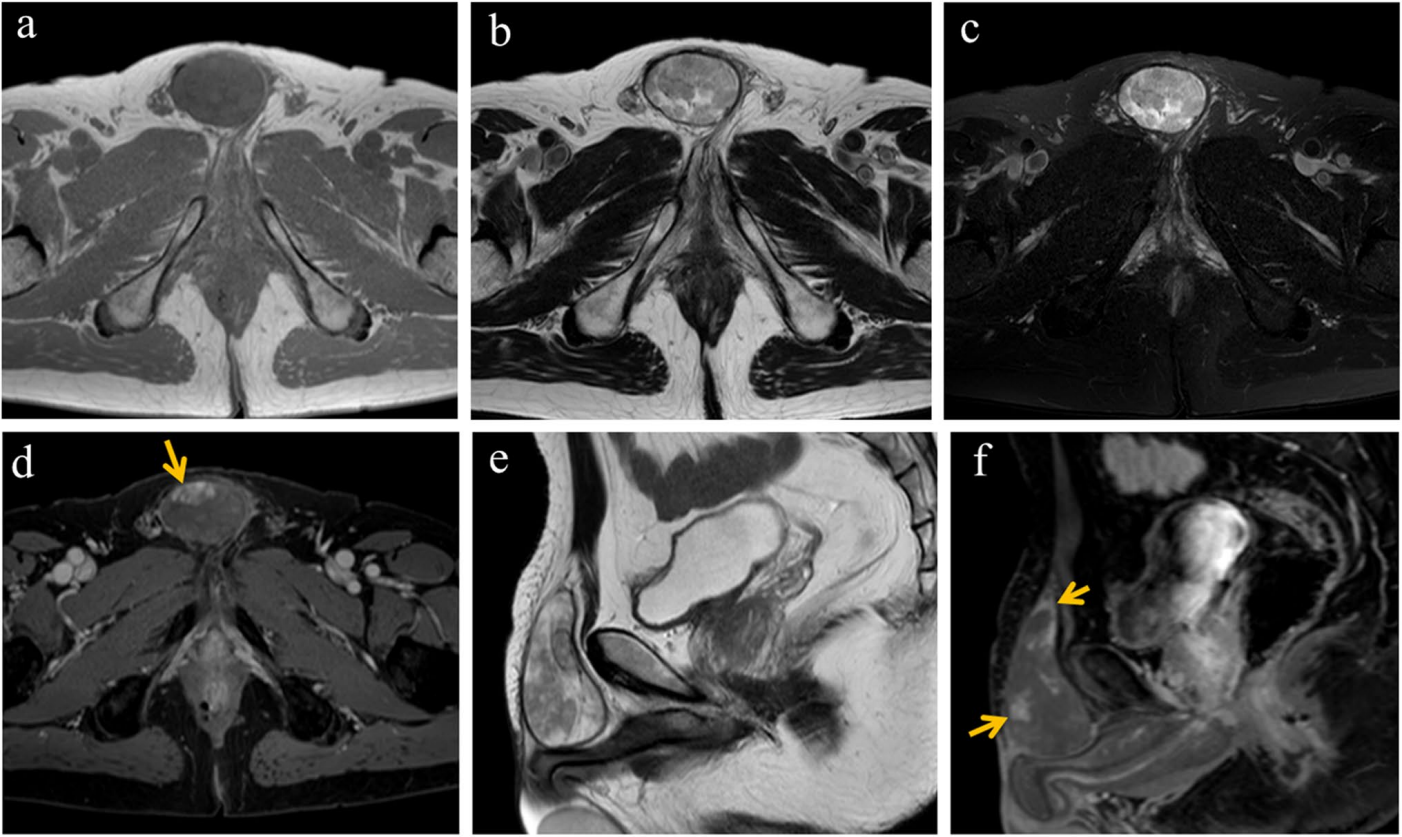
MRI findings of IgG4-related disease. Axial T1WI (**a**) shows low-intensity signals. T2WI (**b**, **e**) and fat-saturated T2WI (**c**) show an intermediate or hyperintensity with a low-signal capsule. Gadolinium-enhanced fat-saturated T1WI (**d**, **f**) shows heterogeneous enhancement (arrows) in the capsule and small areas inside the tumor. MRI, magnetic resonance imaging; T1WI, T1-weighted images; T2WI, T2-weighted images

Further, 1.5 T MRI findings revealed a subcutaneous mass with isointensity compared to the muscle on T1-weighted imaging (WI) (Fig. 1a) and a heterogeneous intermediate or hyperintensity with a low-signal capsule on T2WI (Fig. 1b, e) and fat-suppressed T2 (Fig. 1c). Gadolinium-enhanced T1WI revealed heterogeneous enhancement in the capsule and small areas with a moderately high signal on T2WI; however, there was poor enhancement in areas with intermediate signal intensity on T2WI (Fig. 1d, f). No inguinal lymphadenopathy was observed. Therefore, a poorly contrast-enhanced mass with a capsule, such as myxoma or epidermoid cysts with modifications, was suspected. In addition, a core needle biopsy with 14G suggested a myxoma.

**Fig. 2:**
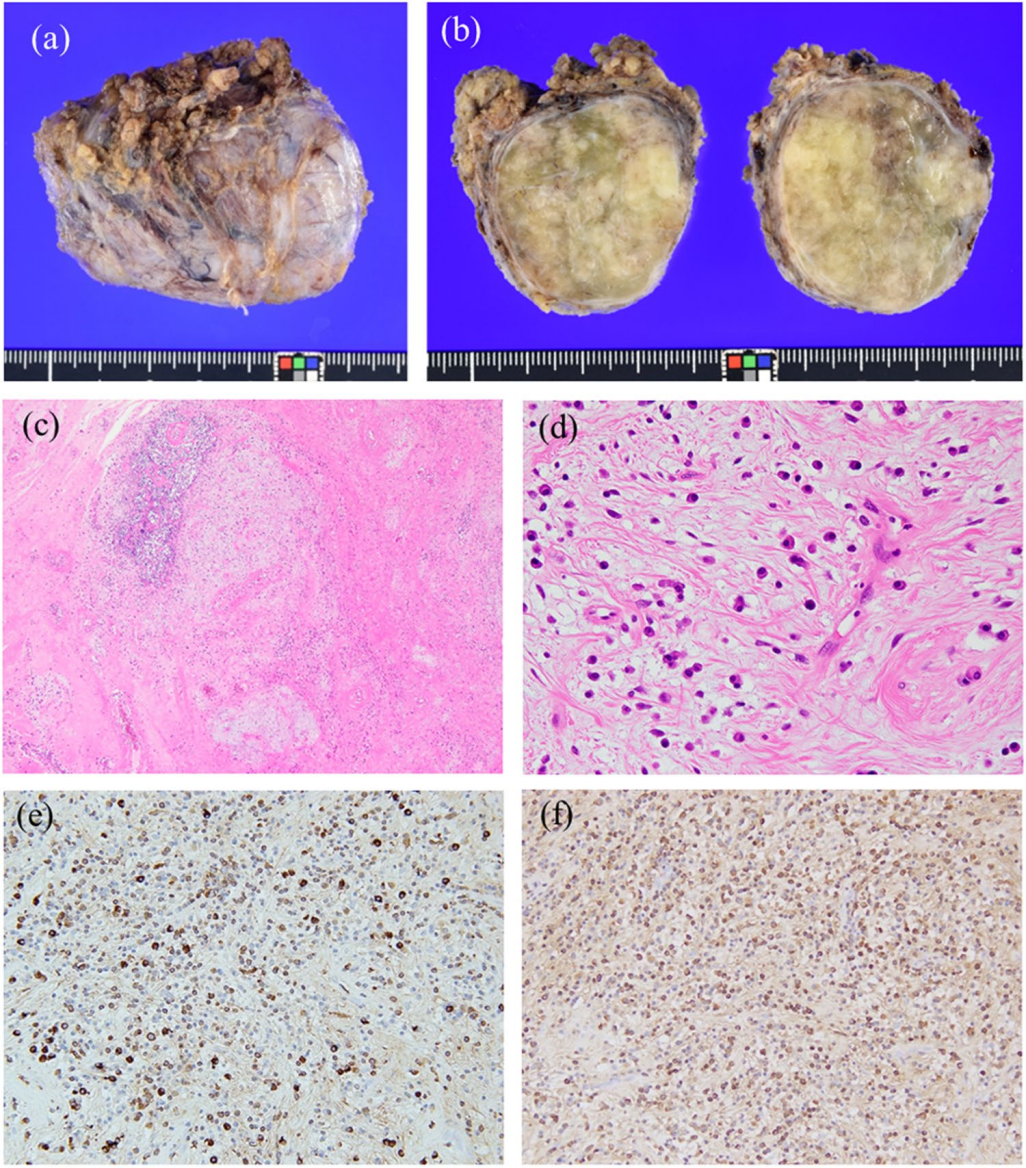
Pathological features of IgG4-related disease. Macroscopically, the tumor is well-defined by the fibrous capsular tissue, and the inside mass is composed of solid and/or transparent elements (**a**, **b**). Histopathologically, intratumoral findings show myxedematous degeneration, fibrosis, as well as predominantly plasmacytic infiltration [Original magnification, × 40 (**c**) and × 100 (**d**); hematoxylin and eosin stain]. The IgG4/IgG ratio is more than 80% [Original magnification, × 100; IgG4 (**e**) and IgG (**f**) immunostain]. H&E, hematoxylin and eosin

We recommended surgery, but the patient declined because of the benign nature of the tumor. One year later, the size of the mass had not changed; however, the patient decided to undergo surgery, which was performed through a transverse incision. The mass and surrounding fat were resected. Macroscopically, the tumor was well-bordered by fibrous capsular tissue, and the inside of the mass showed a solid tumor with a yellow-white to green color (Fig. 2a, b). No necrosis was observed (Fig. 2b). Histologically, the periphery of the mass was bordered by collagen fibers of various thicknesses, and the foci of lymphocytes were scattered. Intratumoral findings showed marked edema and mucous degeneration, in addition to the sparse proliferation of spindle-shaped cells, fibrosis, and prominent infiltration of plasma cells, lymphocytes, mast cells, and macrophages (Fig. 2c, d). The immunohistochemistry findings were as follows: positive for focally positive for CD31; negative for AE1/AE3, CAM5.2, S-100, desmin, SMA, CD99, MDM2, CDK4, β-catenin, STAT6, and ALK; and a Ki67 labeling index < 1%. The kappa/lambda ratio of the light chain was not restricted. The IgG4/IgG ratio was > 80% (Fig. 2e, f); therefore, a final diagnosis of IgG4-RD was made. Three months after surgery, no recurrence was observed.

## Discussion

In the last decade, IgG4-RD has been established as a chronic and progressive autoimmune disorder [4]. IgG4-RD can occur in any organ of the body, with 11 typical organs currently known: pancreas, biliary tract, salivary gland, lacrimal glands, orbit, lung, aorta, retroperitoneum, kidney, pachymeninges, and thyroid [1]. Classification criteria for four phenotypes have recently been suggested as follows: type 1, pancreato-hepato-biliary; type 2, retroperitoneal fibrosis and/or aortitis; type 3, head and neck-limited disease; and type 4, classic Mikulicz inflammation of the organs that can cause type 1 autoimmune pancreatitis in the pancreas or sclerosing cholangitis in the bile ducts [4]. In contrast, IgG4-RD rarely occurs in the bone and soft tissue (subcutaneous and deep layers). To the best of our knowledge, only two cases have been reported in the subcutaneous tissue [3, 6]. The disease predominantly affects middle-aged men and is less likely to affect young children. Many of the cases have been reported from Japan, with an estimated incidence of 0.28–1.08 per 100,000 population and 336–1300 new cases yearly [8].

The three key characteristics for the diagnosis of IgG4-RD include the following: 1) mass or nodules in one or more organs; 2) serum IgG4 level > 135 mg/dL; and 3) presence of lymphoplasmacytic inflammation with prominent IgG4-positive cells [1]. Caution is required regarding the interpretation of serum IgG4 levels, which are biomarkers of disease activity rather than diagnostic markers [1]. In cases of rare presentation, such as ours, serum IgG4 levels are often not measured; therefore, the diagnosis is dependent on pathological findings, such as an IgG4-positive cell count > 10/HPF or a ratio of IgG4-positive cells/IgG cells > 40%. In addition, IgG4 findings in IgG4-RD may overlap with those in chronic granulomatosis [1, 7]. Lymphadenopathy is often seen as a clinical manifestation of IgG4-RD, being reported in 30–55% of cases. Swollen lymph nodes are usually small and asymptomatic; however, lymphadenopathy may cause symptoms due to the compression of adjacent structures. The timing of confirmation of lymphadenopathy varies. Recently, lymphadenopathy has been described as a poor prognostic marker. However, we must pay attention to lymphadenopathy, which has a broad differential diagnosis ranging from benign to malignant diseases [9]. Treatment of both the active and symptomatic disease stages is required to avoid irreversible organ damage. As the first choice, glucocorticoids are recommended; however, in cases with no improvement in symptoms or relapse after the discontinuation of steroids, disease-modifying antirheumatic drugs or rituximab may be effective [4].

In general, there is no doubt regarding the usefulness of MRI in the diagnosis of soft tissue tumors, as in our case [6]; however, it is often difficult to distinguish IgG4-RD from masses, including tumors and inflammation, using imaging techniques alone [5, 10]. In fact, some reviews have reported that MRI showed low intensity on T1WI but inconsistent intensity (low to iso-intensity) on T2WI with variable enhancement [5, 10]. These nonspecific MRI findings pose a diagnostic challenge in IgG4-RD due to the difficulty in distinguishing it from neoplasms [5]. Interestingly, the MRI features in two subcutaneous IgG4-RD cases were reported as follows: 1) subcutaneous layer with linear fascial extension (tail sign), as seen in myxofibrosarcoma and undifferentiated sarcoma; 2) unclear border; and 3) heterogeneous enhancement, which is also observed in sarcoma [3, 6]. These features are similar to those observed in the deep layers of soft tissues [2]. In contrast, the MRI findings of the present case showed a clear margin with a capsule, a negative tail sign, and little enhancement, suggesting a benign tumor such as an epidermoid cyst, myxoid tumors, or vascular malformation. Therefore, because it is impossible to differentiate subcutaneous IgG4-RD from sarcoma using MRI, histopathological diagnosis is crucial.

Although histopathological examination plays a central role in overcoming the limitations of diagnostic imaging, obtaining a differential diagnosis can sometimes be challenging [4]. For example, in lung IgG4-RD, differential diagnoses include inflammatory myofibroblastic tumor, nodular lymphoid hyperplasia, lymphoma, interstitial pneumonia, and granulomatosis with polyangiitis [7]. Distinguishing it from an inflammatory myofibroblastic tumor can be particularly challenging due to the overlap in the number of IgG4-positive cells and IgG4/IgG ratio. Therefore, identifying ALK-positive findings through immunohistochemistry or fluorescent in situ hybridization is crucial for accurately diagnosing an inflammatory myofibroblastic tumor [7]. In our case, the biopsy specimen was suspected to be a myxoma. One reason for this was that the needle biopsy sample was collected from a myxoid-rich lesion in a small quantity; however, at that time, the pathological diagnosis was deemed correct because the MRI findings were consistent with myxoma. Therefore, it is important to consider that the appropriate process with imaging and biopsy may not always lead to an accurate diagnosis. The different natures of the fibrous capsule and myxoid matrix make the imaging findings more complex, and IgG4-RD cannot be differentially diagnosed preoperatively. Notably, positive IgG4 cells were found in the capsule.

In conclusion, we report a rare case of subcutaneous IgG4-RD that was challenging to diagnose. The imaging findings lacked typical features, which could have resulted in a wide range of differential diagnoses, from benign to malignant. In addition, some cases are difficult to diagnose, even with histopathology; therefore, a careful diagnosis must be made based on comprehensive findings, and the physician must remain vigilant to this disease.
